# Comprehensive analysis of circRNA expression profiles and circRNA-associated competing endogenous RNA networks in IgA nephropathy

**DOI:** 10.7717/peerj.10395

**Published:** 2020-12-03

**Authors:** Haiyang Liu, Di Liu, Yexin Liu, Ming Xia, Yan Li, Mei Li, Hong Liu

**Affiliations:** Department of Nephrology, The Second Xiangya Hospital of Central University, Hunan Key Laboratory of Kidney Disease and Blood Purification, Changsha, Hunan, China

**Keywords:** IgA nephropathy, Circular RNA, Peripheral blood mononuclear cell, Competing endogenous RNA network

## Abstract

**Background:**

Immunoglobulin A nephropathy (IgAN) is immune-mediated primary glomerulonephritis, which is the most common reason leading to renal failure worldwide. The exact pathogenesis of IgAN is not well defined. Accumulating evidence indicates that circular RNAs (circRNAs) play crucial roles in the immune disease by involving in the competing endogenous RNA (ceRNA) network mechanism. At present, the studies of the circRNA profiles and circRNA-associated ceRNA networks in the IgAN are still scarce. This study aimed to elucidate the potential roles of circRNA-associated ceRNA networks of peripheral blood mononuclear cells (PBMCs) in IgAN patients

**Method:**

CircRNA sequencing was used to identify the differential expressed circRNAs (DEcircRNAs) of PBMCs in IgAN and healthy controls; limma packages from data sets GSE25590 and GSE73953 in the Gene Expression Omnibus (GEO) database, were used to identify differentially expressed micro RNAs (miRNAs) and message RNAs (mRNAs). A circRNA-miRNA-mRNA ceRNA network was constructed to further investigate the mechanisms of IgAN. Then, GO analysis and KEGG enrichment analyses were used to annotate the genes involved in the circRNA-associated ceRNA network. Further, Protein-protein interaction (PPI) networks were established to screen potential hub genes, by using Search Tool for the Retrieval of Interacting Genes/Proteins (STRING). Last, a quantitative real-time polymerase chain reaction (qRT-PCR) was applied to verify the hub genes in the ceRNA network.

**Result:**

A total of 145 circRNAs, 22 miRNAs, and 1,117 mRNAs were differentially expressed in IgAN compared with controls (*P* < 0.05). A ceRNA network was constructed which contained 16 DEcircRNAs, 72 differential expressed mRNAs (DEmRNAs) and 11 differential expressed miRNAs (DEmiRNAs). KEGG pathway enrichment analysis illustrated the underlying biological functions of the ceRNA-associated genes, such as Nitrogen compound metabolic process, COPII-coated ER to Golgi transport vesicle, CAMP response element protein binding process (*P* < 0.01); meanwhile, Hepatitis B, GnRH signaling, and Prion disease were the most significant enrichment GO terms (*P* < 0.01). PPI network based on STRING analysis identified 4 potentially hub genes. Finally, Ankyrin repeat and SOCS box containing 16 (ASB16), SEC24 homolog C, COPII coat complex component (SEC24C) were confirmed by qRT-PCR (*P* < 0.05) and were identified as the hub genes of the ceRNA network in our study.

**Conclusion:**

Our study identified a novel circRNA-mediated ceRNA regulatory network mechanisms in the pathogenesis of IgAN.

## Introduction

IgA nephropathy (IgAN) has a global incidence exceeding 1.5 per 100,000 persons, which is the most common type of glomerulonephritis worldwide (Wyatt & Julian, 2013). The clinical manifestation often presents as macroscopic hematuria following an upper respiratory or gastrointestinal infections. Diagnosis of IgAN is exclusively identified by renal biopsy with characteristics of predominant IgA deposition in the glomerular mesangium (Wyatt & Julian, 2013), and the 10-year risk of end-stage renal disease varies widely from 5% to 60% (Reich et al., 2007). In exploring of pathogenesis in IgAN, many mechanisms including the four-hit hypothesis had been proposed in academic literature: the unknown upstream inducing the production of galactose-deficient IgA1(Gd-IgA1), which were identified as auto-antigen and targeted by auto-antibody, formatted as immune complex in the circulation, deposited in kidney thus resulting in following renal injuries (Suzuki et al., 2011). Noticeably, recurrence of IgAN is 50% over 5 years after kidney transplantation (Mulay, vanWalraven & Knoll, 2009), while it is reported that several IgAN cases experienced a recovery after bone marrow transplantation (Park et al., 2008), suggesting IgAN appears to be a systemic immune disease in which the kidneys are damaged as innocent bystanders (Wyatt & Julian, 2013).

Recently, circular RNAs (circRNAs) have aroused attention as one of the factors correlated with immune response; they are special types of single-stranded non-coding RNA which has a closed feature and without 3′ poly (A) and 5′-cap structure (Chen, 2020; Yan & Chen, 2020). CircRNA is formed by the back-splicing of pre-mRNAs processing, in which a downstream 5′ splice site is joined to an upstream 3′ splice site in reverse order across an exon or exons. An investigator recently discovered that aftef a B cell was infected with Kaposi’s sarcoma herpesvirus, hundreds of differentially expressed human circRNAs were identified, which suggests those newly expressed circRNAs may work as an antiviral mechanism by suppressing crucial viral genes (Tagawa et al., 2018). The expression of circRNAs is usually stable as it is resistant to exonucleases, and largely exported to the cytoplasm (Chen, 2020). The harboring miRNA recognition elements (MREs) of circRNAs could competitively bind to certain miRNAs, and regulate miRNA-mediated downstream target gene silencing at the post-transcriptional level, thus participating in the manipulation process of the target genes and described as involved in the competing endogenous RNA (ceRNA) hypothesis (Thomson & Dinger, 2016).

The ceRNA hypothesis has been demonstrated to be involved in kidney disease: for example, the novel_circ-0004153/rno-miR-1443p/Gpnmb ceRNA relationship has been reported involved in acute kidney injury of a rat model (Cheng et al., 2019); circHLA-C could function as a sponge to decoying miRNA-150, then promoted renal fibrosis by regulating fibrosis-associated genes in lupus nephritis (Luan et al., 2018). However, studies of the circRNA profiles and circRNA-associated ceRNA networks in the IgAN are still scarce; therefore, exploring the expression of non-coding RNAs including circRNAs and miRNAs may bring potential opportunities for understanding the mechanism of IgAN.

**Figure 1:**
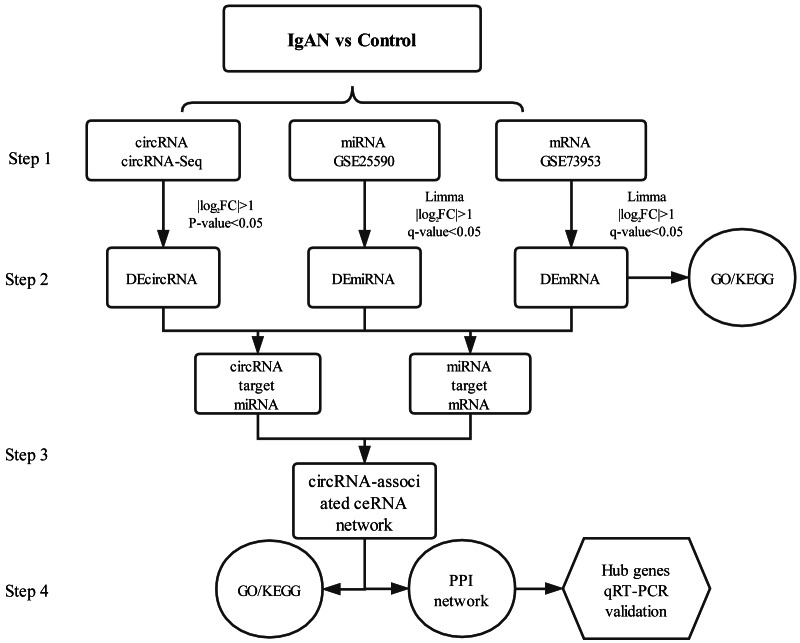
Main steps of the construction of the circRNA-associated ceRNA network in IgAN. Step 1: The circRNA expression profiles of PBMCs in IgAN patients and healthy controls were explored by high-throughput sequencing, expression profiles of miRNAs and mRNAs were obtained from the data sets in the GEO database. Step 2: Significantly differential circRNAs, miRNAs and mRNAs were identified, the GO and KEGG enrichment analysis were conducted to reveal the functions of differential expressed mRNAs. Step 3: The ceRNA interaction relationships were predicted using online tools, then constructed the ceRNA network. Steps 4: Functional analysis of the related genes in the ceRNA network. Identification hub genes of the ceRNA network and validate the expression with qRT-PCR. IgAN: IgA nephropathy. DEcircRNA: differentially expressed circularRNA. DEmiRNA: differentially expressed microRNA. DEmRNA: differentially expressed mRNA. PBMCs: peripheral blood mononuclear cells. CeRNA: competing endogenous RNA. GO: Gene Ontology. KEGG: Kyoto Encyclopedia of Genes and Genomes.

PBMCs are blood cells with round nuclei that encompass a heterogeneous cell population (with 70–90% T cells, B cells, and NK cells) (Sallustio et al., 2019), which originate from hematopoietic stem cells that reside in the bone marrow. In this study, PBMCs from IgAN patients and healthy controls were collected and sequenced with next-generation technology and, with combined analysis of the genes expression profiles in the GEO database, a circRNA associated ceRNA network of IgAN was established (Fig.1) with the aim of comprehensively investigating the potential circRNA related molecular mechanism in IgAN.

## Materials & Methods

### PBMCs collection and RNA preparation

This research was approved by the Ethics Committee of the Second Xiangya Hospital (IRB2018-S095). Participants were from The Second Xiangya Hospital, and all of them had provided signed informed consent. The diagnosis of IgAN was based on renal biopsy with the deposition of IgA in the glomerular mesangium (Wyatt & Julian, 2013), patients with any other systemic diseases or secondary IgAN were excluded. Peripheral blood from 3 IgAN patients and 3 healthy controls were centrifuged at 2,000 rpm for 10 min, PBS was added to diluted the remaining blood, by Ficoll-Hypaque (GE Healthcare) gradient (2,000 rpm for 30 min) to isolate the PBMCs by density separation. The isolated PBMCs were used for RNA isolation. Then, total RNA was extracted using Trizol reagent (Invitrogen, Carlsbad, CA, USA) following the manufacturer’s procedure. The total RNA quantity and purity were analyzed using Bioanalyzer 2100 and RNA 6000 Nano LabChip Kit (Agilent, Santa Clara, CA, USA) with RNA integrity number > 7.0. Approximately 5 ug of total RNA were used to deplete ribosomal RNA according to the Ribo-Zero™ rRNA Removal Kit (Illumina, San Diego, USA). After removing ribosomal RNAs, the remaining RNAs were fragmented into small pieces using divalent cations under high temperatures. Then the cleaved RNA fragments were reverse-transcribed to create the cDNA, which was next used to synthesized U-labeled second-stranded DNAs with E. coli DNA polymerase I, RNase H and dUTP. An A-base is then added to the blunt ends of each strand, preparing them for ligation to the indexed adapters. Each adapter contains a T-base overhang for ligating the adapter to the A-tailed fragmented DNA. Single-or dual-index adapters are ligated to the fragments, and size selection was performed with AMPureXP beads. After the heat-labile UDG enzyme treatment of the U-labeled second-stranded DNAs, the ligated products were amplified with PCR with the following conditions: initial denaturation at 95 °C for 3 min; 8 cycles of denaturation at 98 °C for 15s, annealing at 60 °C for 15s, and extension at 72 °C for 30s; and then final extension at 72 °C for 5 min. The average insert size for the final cDNA library was 300 bp (±50 bp).

### CircRNA sequencing and microarray data download

First, we performed the paired-end sequencing (PE150) for the library on Illumina Hiseq 4000 (LC Science Co., LTD., Hangzhou, China) following the vendor’s recommended protocol. Cutadapt (Martin, 2011) was used to remove the reads that contained adaptor contamination, low quality bases and undetermined bases. Then sequence quality was verified using FastQC (http://www.bioinformatics.babraham.ac.uk/projects/fastqc/). We used Bowtie2 (Langmead & Salzberg, 2012) and Hisat2 (Kim, Langmead & Salzberg, 2015) to map reads to the genome of Homo sapiens (GRCh38). Remaining reads (unmapped reads) were still mapped to genome using tophat-fusion (Kim & Salzberg, 2011). CIRCExplorer2 (Zhang et al., 2014; Zhang et al., 2016) was used to de novo assemble the mapped reads to circRNAs at first. Then, back splicing reads were identified in unmapped reads by tophat-fusion. All samples generated unique circular RNAs ( step1: the two ends of splice sites must be GU/AG; step 2: mismatch ≤2; step 3: back spliced junctions read ≥1; step 4: the distance between two splice sites on the genome is not more than 100 kb).

We next performed an electronic search in GEO (http://www.ncbi.nlm.nih.gov/geo/) database using the keywords “IgA nephropathy, IgA nephritis, or Berger’s disease” to identify studies involving samples with PBMCs from IgAN patients. Studies were included if they met the following criteria: (1) patients were diagnosed with biopsy-proven IgAN; (2) case-control studies and the number of cases and controls in each dataset must be ≥2; (3) all datasets were genome-wide; (4) complete microarray raw data were available. We excluded any animal or duplicated studies. The whole-genome raw expression data of included studies were downloaded from the GEO dataset. Therefore, the GSE73953 (mRNA) and GSE25590 (miRNA) microarray datasets that contain IgAN’s PBMC related information were selected. GSE73953 including 15 IgAN patients, 2 healthy controls (Nagasawa et al., 2016), which was based on the GPL4133 platform (Agilent-014850 Whole Human Genome Microarray 4x44K G4112F). GSE25590 contained seven IgAN patients and seven matched healthy samples (Serino et al., 2012) and was based on the GPL7731 platform (Agilent-019118 Human miRNA Microarray 2.0 G4470B).

### Differential expression analysis of circRNAs, miRNAs, and mRNAs

The unit of measurement for circRNA is Fragment Per Kilobase of exon per Million fragments mapped (FPKM). The differentially expressed circRNAs were selected with log2 (fold change) >1 or log_2_ (fold change) <-1 (|log_2_ (FC)|>1) and with statistical significance (*P*-value < 0.05) by R package–edgeR (Robinson, McCarthy & Smyth, 2010).

The expression status of GSE73953 and GSE25590 were obtained by R-package agimicrorna processing, normalizeBetweenArrays in the Limma package was used to normalize the expression values in the quartile (Ritchie et al., 2015). Finally, log2 transformation was used to obtain standardized expression values. Significance of differential miRNAs and mRNAs expression were defined by —log_2_(FC)—>1, *q*-value <0.05 and *P*-value <0.05. The heat maps were made by Pheatmap software, and the volcano plot was made by Ggplot2 software.

### Function enrichment analysis of the differential expression genes

To investigate the potential function of the differential expression genes (DEGs) in the PBMCs of IgAN, GO (http://www.geneontology.org/) and KEGG (http://www.kegg.jp/) pathway enrichment analysis of the identified DEGs were performed. The identified DEGs were classified in terms of the biological process (BP), molecular function (MF), and cellular component (CC) categories (Ashburner et al., 2000). KEGG analysis was utilized to interpret the potential functions and pathways of the DEGs (Kanehisa et al., 2010).

### CeRNA network construction and functional analysis of the genes in the network

We analyzed the circRNAs and mRNAs which are expressed in significantly different levels between the IgAN patients and the control group. The sequences of circRNAs, miRNAs, and mRNAs were screened to search the potential MREs. We used four databases including miRanda (http://www.microrna.org/microrna/home.do), PITA (http://genie.weizmann.ac.il/pubs/mir07/mir07_dyn_data.html), RNA22 (http://cm.jefferson.edu/rna22/Interactive/) and TarPmiR (http://hulab.ucf.edu/research/projects/miRNA/TarPmiR/) to find miRNAs-circRNAs relationship, only overlapping genes were selected as candidate. Next, we used five kinds of databases including MiRDB (http://mirdb.org/), TargetScan (http://www.targetscan.org/), miRanda, miRMap (https://mirmap.ezlab.org/) and miTarBase (http://mirtarbase.mbc.nctu.edu.tw/) to screen mRNAs corresponding targets of miRNAs, and retained the interaction genes which are identified at least four of the databases. The circRNA-miRNA-mRNA regulatory network was constructed using a combination of circRNA-miRNA and miRNA-mRNA interaction. Finally, the network was visualized and mapped using Cytoscape v3.7.0 software. All of the DEGs coming from co-expression and prediction ceRNA network were united for genes functional annotation and enrichment analysis, to investigate the potential molecular mechanism of the genes in IgAN.

### Protein–protein Interaction network analysis and qRT-PCR of the hub genes

To assess the interactions between DEGs in the ceRNA network, we used the Search Tool for the Retrieval of Interacting Genes (STRING, https://string-db.org/) online tool, which can provide comprehensive interactions among proteins and genes, and established a Protein-Protein Interaction (PPI) network of our ceRNA network. A Required Confidence (combined score) >  0.7 was used to visualize the established PPI network. Then we used Cytoscape to analyze the topology of genes in the PPI network, the central proteins in the network are found by using the scale-free nature of the interaction PPI network. Also, 4 IgAN patients and 4 healthy controls in the Second Xiangya Hospitals that signed informed consent, were recruited for qRT-PCR validation of the expression of hub genes. The way of PBMCs collection and RNA extraction were mentioned previously, the qRT-PCR reaction was performed using the UltraSYBR Mixture (Cwbiotech, China), in the Pikoreal PCR Detection System (Thermo, USA) with the following conditions: 95 °C for 10 mins, then followed by 40 cycles of 95 °C for 15s and 60 °C for the 30s. The quantitative primers of Ankyrin repeat and SOCS box containing 16 (ASB16) , major histocompatibility complex, class I, B (HLA-B), tripartite motif containing 21(TRIM21), SEC24 homolog C, COPII coat complex component (SEC24C) , were designed and synthesized by Sangon Biotech (Sangon Biotech, China) and are listed in Table S1, β-actin was used as the house-keeping gene for normalization, qRT-PCR relative fold change results were calculated using the 2-△△Ct method.

## Results

### Overview of circRNA-Seq and identification of DEcircRNAs

After the removal of low-quality reads, adapters, poly-N>5%, and other contaminant-containing reads from the raw data, clean reads of circRNA-sequencing (circRNA-seq) were obtained.

**Figure 2:**
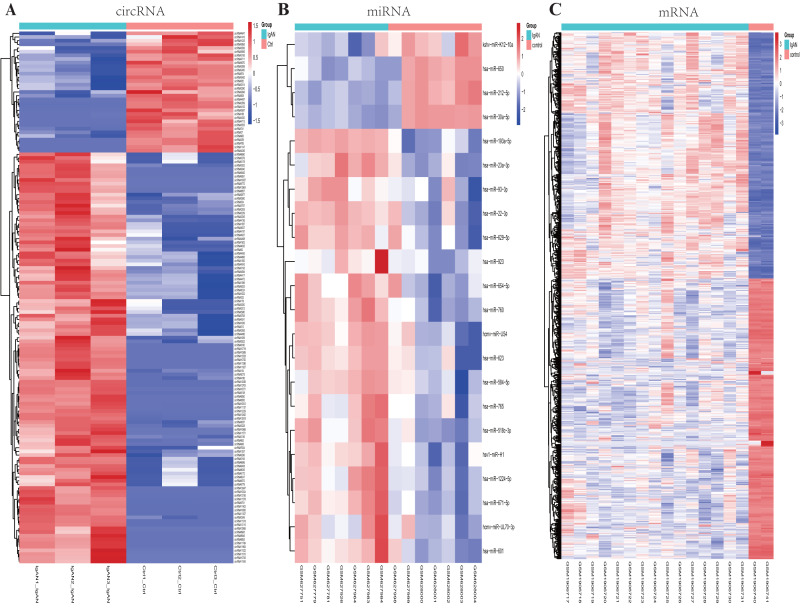
Heatmaps of differential expressed circRNAs, miRNAs and mRNAs. Cluster analysis of expression of (A) circRNAs, (B) miRNAs and (C) mRNAs. Red and blue: increased and decreased expression, respectively. Columns represent different samples, rows indicates different genes. Significantly differential circRNA were defined by |log_2_ (FC)|>1 and *P*-value < 0.05, the differential expression miRNAs and mRNAs were defined by |log_2_ (FC)|>1, *P*-value < 0.05 and *q*- value <0.05.

In the present study, a total of 16,940 circRNA transcripts were identified, among them, 145 circRNAs were differentially expressed in the IgAN patients compared to the healthy controls, including 112 up-regulated and 33 down-regulated circRNAs. The basic characteristic of the top 10 DEcircRNAs are listed in Table 1, the most up-regulated circRNA was hsa_circ_0038725 with 6.03 log_2_(FC), and the most down-regulated circRNA was circRNA11137 (gene symbol: EMB) with -6.01 log_2_(FC), those DEcircRNAs were used for the subsequent analysis (Table S2). A heat-map of DEcircRNAs was illustrated in Fig. 2A.

**Table 1:** The ten most dysregulated circRNA transcripts between IgAN patients and healthy controls.

Accession	CricBase	Gene symbol	Chromosome	Strand	Regulation	log _2_(FC)	*P*-value
circRNA9482	hsa_circ_0038725	IL4R	Chr16	+	Up	6.03134	0.01027
circRNA11137	novel_circ	EMB	Chr5	–	Up	6.01153	0.00447
circRNA12492	novel_circ	SP140L	Chr2	+	Up	5.98330	0.00015
circRNA4713	novel_circ	TLN1	Chr9	–	Down	5.69939	0.02184
circRNA10024	novel_circ	RPS6KA5	Chr14	–	Up	5.59830	0.00903
circRNA8550	hsa_circ_0028670	TAOK3	Chr12	–	Up	5.46949	0.00979
circRNA10745	hsa_circ_0007612	ORC5	Chr7	–	Up	5.16406	0.03555
circRNA11518	novel_circ	HERC3	Chr4	+	Up	5.13206	0.00143
circRNA4236	hsa_circ_0004893	PTP4A2	Chr1	+	Down	4.74473	0.00931
circRNA11045	hsa_circ_0009096	UTRN	Chr6	+	Up	4.49705	0.03553

### Identification of DEmiRNAs and DEmRNAs

**Figure 3:**
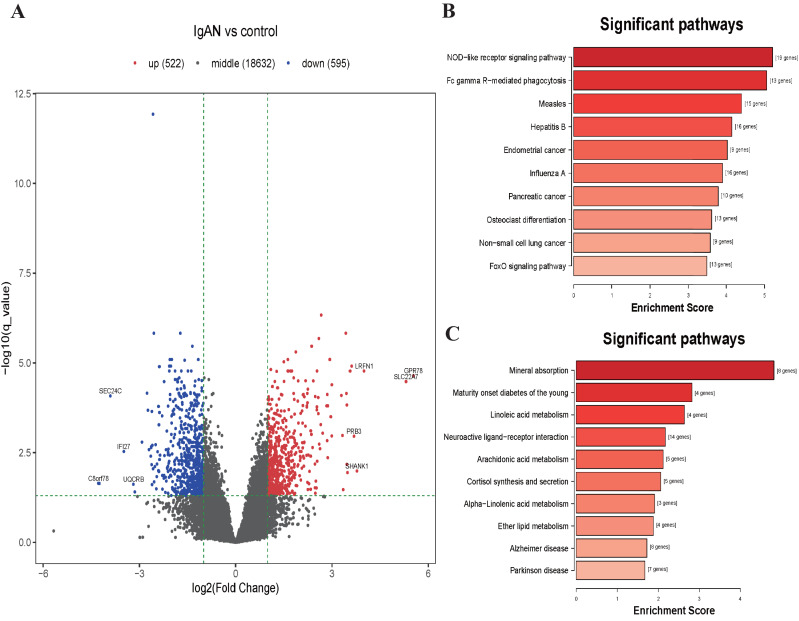
Volcano plot and top 10 enrichment KEGG pathways for DEGs. (A) Volcano plot for the DEGs in data set GSE73953. The *x*-axis indicates the log_2_(FC), and the *y*-axis indicates the log10 (*q*-value). The red dots represent up-regulated genes, and the blue dots represent down-regulated genes. The black dots represent the genes expressed without significant differences. (B) Top 10 enrichment KEGG pathways for down-regulated DEGs, and (C) up-regulate DEGs, the *x*-axis shows the names of the pathway names, the *y*-axis shows enrichment score. DEGs: differentially expressed genes.

A total of 22 DEmiRNAs were screened from the GSE25590 dataset, including 18 up-regulated and 4 down-regulated miRNAs in IgAN patients (Table S3). In the GSE73953 dataset, a total of 1117 DEmRNAs were identified, including 522 up-regulated and 595 down-regulated mRNAs in IgAN patients (Table S4). The DEmiRNAs and DEmRNAs between IgAN patients and controls are illustrated as heat-map in Fig. 2BC. The statistical analysis of the differential expressed circRNAs, miRNAs and mRNAs were summarized in Table 2. A volcano plot was utilized to visualize the statistical significance of DEGs between the IgAN patients and the controls (Fig. 3A).

**Table 2:** Statistical analysis of differentially expressed circRNAs, miRNAs and mRNAs.

Data resource	Type of RNAs	Samples		Platforms	Differential expression		Threshold		Max log_2_(FC)	
		IgAN	Control		Up	Down	log2(FC)	*P*-value	Up	Down
RNA-seq	circRNAs	3	3	GPL20301	112	33	1	0.05	6.03	−6.01
GSE25590	miRNAs	7	7	GPL7731	18	4	1	0.05	2.74	−1.99
GSE73953	mRNAs	15	2	GPL4133	522	595	1	0.05	5.54	−4.27

### Pathway enrichment analysis of the DEGs

KEGG analysis illustrated those down-regulated DEGs were mainly enrichment in the NOD-like receptor signaling pathway (hsa04621, 19 genes were enriched with *P* < 10^−6^), Fc gamma Receptor-mediated phagocytosis (hsa04666, 13 genes were enriched with *P* < 10^−6^), Measles pathway (hsa05162, 15 genes were enriched with *P* < 10^−5^), etc (Fig. 3B). Those upregulated DEGs were mainly enriched in the Mineral absorption pathway (hsa04978, 8 genes were enriched with *P* < 10^−5^), Maturity onset diabetes of the young pathway (hsa04950, 4 genes were enriched with *P* < 10^−4^), Linoleic acid metabolism pathway (hsa00591, 4 genes were enriched with *P* < 10^−4^), etc (Fig. 3C). The results of GO analysis shows that the down-regulated DEGs were enriched in functions associated with Leukocyte activation involved in immune response (GO:0002366, 55 genes were enriched with *P* <10^−15^), Cytoplasm (GO:0005737, 382 genes were enriched with *P* < 10^−16^), Identical protein binding (GO:0042802, 80 genes were enriched with *P* < 10^−5^). The upregulated DEGs were enriched in function associated with Cellular response to copper ion (GO:0071280, 10 genes were enriched with *P* < 10^−11^), Integral component of postsynaptic specialization membrane (GO:0099060, 8 genes were enriched with *P* < 10^−5^), Hormone activity (GO:0005179, 8 genes were enriched with *P* < 10^−4^) (Table S5 and Fig. S1).

### Construction of the ceRNA network and functional annotation of genes in ceRNA network

**Figure 4:**
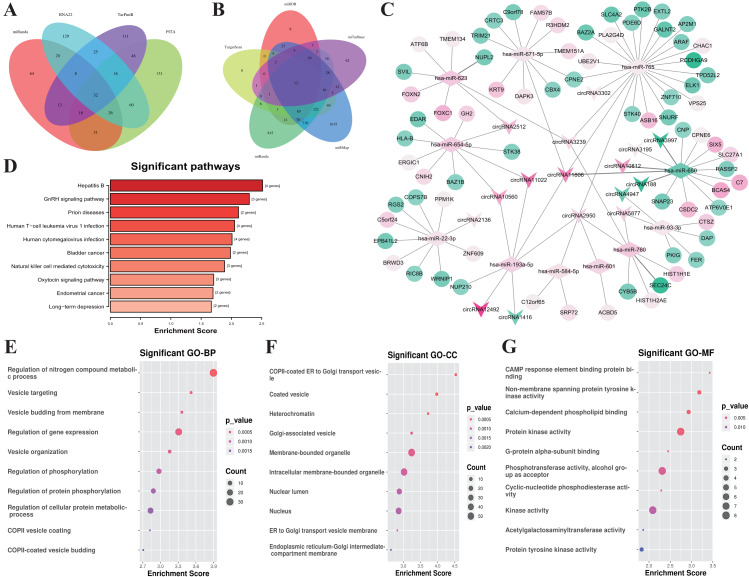
Construction of the ceRNA network and functional analysis of the genes in the network. (A) Venn diagram showed the online tools were used in circRNA-miRNA corresponding relationship prediction and the (B) miRNA-mRNA corresponding prediction. (C) The circRNA-associated ceRNA network. The round nodes represent DEmRNAs, the rhombus represent DEmiRNAs, the v-type denote DEcircRNAs. The up or down-regulated genes are represented in pink and green, respectively, the darker color indicates the larger absolute value of Log_2_(FC). (D) Top 10 enrichment KEGG pathways for the related genes in the ceRNA network, the *x*-axis shows the names of the pathway, the *y*-axis shows enrichment score. (E) Top 10 GO enrichment process categorized by biological process (BP), (F) molecular function (MF), and (G) cellular component (CC), red indicates higher enrichment, the sizes of the dots represent the numbers of genes in each GO category.

According to the ceRNA hypothesis, the members of ceRNA (circRNAs, miRNAs, and mRNAs) compete for the same miRNA response elements (MREs) to regulate each other. To establish a circRNA-miRNA-mRNA ceRNA network, we found 32 DEmiRNAs-DEcircRNAs pairs according to the circRNAs-miRNAs corresponding relationship, which overlapped in 4 databases including miRanda, PITA, RNA22, and TarPmiR (Fig. 4A).

Then, we used five kinds of databases including MiRDB, TargetScan, miRanda, miRMap and miTarBase to identify mRNAs corresponding targets of miRNAs, and 12 DEmRNAs-DEmiRNAs relationship pairs which are overlapping at least 4 of the databases. Overlapping datasets were visualized using Venn diagrams (Fig. 4B).

Finally, we constructed a ceRNA network based on 16 circRNA nodes, 11 miRNA nodes, and 72 mRNA nodes (Fig. 4C). These RNA interactions may serve as a novel perspective for exploring the underlying mechanism of IgAN. More details are listed in Table S6.

All of the related genes coming from the established ceRNA network were united for genes functional annotation enrichment analysis to investigate the potential roles of these differentially expressed circRNAs and mRNAs (Table S7). The KEGG analysis showed that the pathway of genes in the network were mostly related with Hepatitis B (hsa05161, 4 genes were enriched with *P* = 0.003), GnRH signaling pathway (hsa04912, 3 genes were enriched with *P* = 0.005), and Prion disease (hsa05020, 2 genes were enriched with *P* = 0.007) (Fig. 4D). The most significant biological process is the regulation of Nitrogen compound metabolic process (GO:0051171, 35 genes were enriched with *P* < 10^−4^), Vesicle targeting (GO:0006903, 4 genes were enriched with *P* < 10^−4^), Vesicle budding from the membrane (GO:0006900, 4 genes were enriched with *P* < 10^−4^) (Fig. 4E). The most relevant cellular component are COPII-coated ER to Golgi transport vesicle (GO:0030134, 5 genes were enriched with *P* < 10 ^−4^), Coated vesicle (GO:0030135, 7 genes were enriched with *P* < 10^−4^), and Heterochromatin (GO:0000792, 4 genes were enriched with *P* < 10^−3^) (Fig. 4F). CAMP response element binding (GO:0008140, 2 genes were enriched with *P* < 10 ^−3^), Non-membrane spanning protein tyrosine kinase activity (GO:0004715, 3 genes were enriched with *P* < 10 ^−5^), and Calcium-dependent phospholipid binding (GO:0005544, 3 genes were enriched with *P* = 0.001) were the most enrichment molecular function (Fig. 4G).

### Protein–protein Interaction network analysis and validation of the hub genes

**Figure 5:**
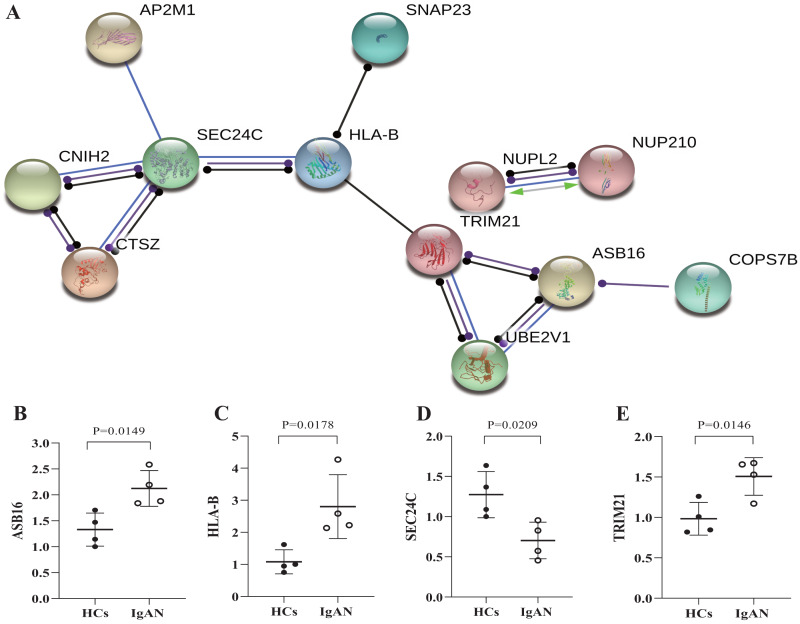
PPI network analysis of ceRNA-associated genes and qRT-PCR validation of the potentially hub genes. (A) PPI network was constructed with genes from the ceRNA network, by using online tool STRING and threshold required confidence (combined score) > 0.7. (B) The relative expression levels of ASB16, (C) HLA-B, (D) TRIM21, (E) SEC24C by qRT-PCR analysis in the PBMCs from HCs and IgAN patients (*n* = 8), β-actin was used as the house-keeping gene for normalization, data were expressed as a ratio of average 2-ΔΔCt, and means ± SD. PPI: Protein-protein interaction. STRING: Search tool for the retrieval of interacting genes/proteins. HCs: healthy controls.

Furthermore, the genes were selected which has potential functional associated (in the STRING database) with other genes in the ceRNA network, and then established PPI networks. The PPI network involving 7 nodes and 4 edges (Fig. 5A). Among then, ASB16, HLA-B, TRIM21, SEC24C were relatively highly connected in the PPI network (associated with more than 3 molecules) and were considered to be the potential hub genes (He & Zhang, 2006). qRT-PCR was utilized to validates those hub genes in the PPI networks (Figs. 5B–5E), as a result, ASB16 was confirmed significant up-regulated (*P* = 0.015) (Fig. 5B) and SEC24C was significantly down-regulated in IgAN patients (*P* = 0.021) (Fig. 5D). This indicates the up-regulated ASB16 and down-regulated SEC24C may play important roles in the pathogenesis and pathological process in IgAN.

## Discussion

In the presenting study, we successfully identified DEcircRNAs in PBMCs of IgAN (*P* < 0.05), combined with the identified DEmiRNAs and DEmRNAs (*P* < 0.05) in the RNA profiles in the GEO database, a ceRNA network was constructed with 16 DEcircRNAs, 11 DEmiRNAs, and 72 DEmRNAs according to the ceRNA hypothesis. The function analysis revealed the genes in the ceRNA network mainly involved in the Nitrogen compound metabolic process, COPII-coated ER to Golgi transport vesicle, CAMP response element protein binding process in the GO term annotation, meanwhile, it’s showed that the Hepatitis B, GnRH signaling pathway, Prion disease and Human T-cell leukemia virus 1 infection were the most enriched KEGG pathway, and interestingly, 3 of them were closely related to the virus infection. The protein interaction analysis revealed that ASB16, SEC24C were considered as hub genes in the ceRNA networks. Therefore, our results have firstly revealed the potential mechanism of circRNAs-associated ceRNA networks in IgAN.

Since the 1990s, many studies have found that competing endogenous circRNAs could work as endogenous miRNA ‘sponges’, which take part in regulating miRNA related downstream gene expression (Chen, 2020). Ongoing investigation illustrated that significant expression of circRNAs was observed in platelets, hematopoietic progenitor cell differentiation into lymphoid and myeloid cells, and been observed they have functionally involved in neuronal function, cell proliferation, and innate immunity. But the pathological function of circRNA is still largely unknown, for instances, rheumatoid arthritis is a chronic and systemic autoimmune disease with unknown etiology (Coutant & Miossec, 2020), multiple groups have independently identified several differentially expressed circRNAs in PBMCs between rheumatoid arthritis patients and healthy controls (Wen et al., 2020; Yang et al., 2019). Similarly, systemic lupus erythematosus (SLE) in one of the autoimmune disease and could also be manifested as glomerulonephritis, several circRNAs including hsa_circ_0044235 have been found to be significantly decreased in PBMCs, and are proposed as potential negative biomarkers for SLE diagnosis (Luo et al., 2019).

In the field of IgAN, several studies have investigated the non-coding RNA in PBMCs. For examples, UDP-N-acetyl-α-d-galactosamine: polypeptide N-acetylgalactosaminyltransferase 2 (GALNT2) could catalysis the attachment of N-acetylgalactosamine (GalNAc) to the serine/threonine of the hinge region of the molecule, which functions in the early step in O-linked glycosylation in IgA1, the significantly down-regulated GALNT2 in the PBMCs thus correlated with the Gd-IgA1 level in IgAN, Serino et al. had reported that miRNA let-7b not only involving influence the expression of GALNT2, it could also play roles as a biomarker for detecting primary IgAN (Serino et al., 2016). In the presenting study, we have first time identified 112 up-regulated and 33 down-regulated circRNAs in IgAN, the interaction regulatory mechanism of the related circRNA have been carefully predicted. Consistent with the literature, the GALNT2 were significantly down-regulated in the PBMCs of IgAN in our study, while the hsa_circ_0070562, hsa_circ_0066719, hsa_circ_0073237 and circRNA3302 were identified as ceRNAs of miR765, therefore could participate in the manipulation the expression of target GALNT2. However, unexpectedly all the 3 circRNAs were up-regulated in the study, therefore didn’t follow the classic circRNA (down in IgAN)-miRNA (up in IgAN)-mRNA (down in IgAN) interaction mechanism, therefore, it remains unknown whether the regulation of GLANT2 in IgAN is influenced by the circRNAs in PBMCs. It’s should be mentioned that Thomson & Dinge used to discuss in 2016 (Thomson & Dinger, 2016), the underlying argument opinion which against the ceRNA hypothesis is that, the change in expression of an individual non-coding RNA may only impact relatively a small fraction of the target mRNA abundance. Therefore, the real mechanism of ceRNA interaction needs further molecular biology experiments, to carefully verify this non-coding factors on the molecules in the disease, such as the previous literature, using knock-out or overexpression method to illustrated a TGF-β/Smad3-interacting-lncRNA inhibits renal fibrogenesis in IgAN (Wang et al., 2018).

Next, we identified the hub genes of the ceRNA network. Normally most proteins interact with only a few other proteins, while a small number of proteins that have many interaction partners (the genes encoding them, namely hub genes) (He & Zhang, 2006), and usually play important roles in the protein network, exploring of the hub genes may bring further insights into the presented ceRNA network. In our study, ASB16 and SEC24C were confirmed by qRT-PCR to have the same gene expression direction as detected by microarray, thus were identified as potentially the hub genes of the ceRNA networks. ASB16 is a member of the Ankyrin repeat and SOCS box-containing (ASB) family of proteins, which may be a substrate-recognition component of E3 ubiquitin-protein ligase complex that mediates the ubiquitination and subsequent proteasomal degradation of target proteins (Liu, Verhaar & Peppelenbosch, 2019); SEC24C is a subunit of COPII complex, which is the coat protein complex responsible for vesicle budding from the endoplasmic reticulum (ER), and plays a role in shaping the vesicle (Subramanian et al., 2019), as well as in cargo selection and concentration, It’s been reported SEC24C were significantly up-regulated during B cell differentiation into a plasma cell and was assumed associated with the antibody-producing related preprocessing (Kirk et al., 2010).

In our study, the functional analysis of the DEGs in the PBMCs according to the genes profiles GSE73953 had shown, the NOD-like receptor signaling pathway, Fc gamma R-mediated phagocytosis, Mineral_absorption pathway were the most enriched pathway. In another study that had comprehensively analyzed 3 microarray data of PBMC in IgAN (Liu et al., 2019), the KEGG enrichment pathway analysis was confirmed with enriched in human T-cell leukemia virus 1 infection, but also including proteoglycans in cancer and intestinal immune network for IgA production. However, noticeably, our study had suggested that the genes in the circRNA-associated ceRNA network were relatively more enriched in virus infection-related pathway. The KEGG analysis indicated that the Hepatitis B, Prion disease, T-cell leukemia virus 1 infection, and cytomegalovirus infection were significantly enriched in the genes of the ceRNA network. It’s well known that viruses could manipulate host gene expression, and among the various mechanism of this pathological process, the most efficient way is a non-coding RNAs strategy (Tycowski et al., 2015). Many kinds of research have revealed that viruses produce non-coding RNAs could serve as miRNA sponges, play as an adaptation to the ceRNA, and increased ceRNA activity (Tycowski et al., 2015). But the mechanism investigation of virus-related ceRNA in IgAN was less explored up till now. In the related literature, the relationship between IgAN and HBV has been often noticed, it’s reported 17–18% of overall IgAN patients in China were HBV carriers (Zhao et al., 2020). Presently it’s unknown that, whether the humoral immune response inducing by HBAg-HBAb immune complex causing the dysregulation of IgA producing or the mesangial cell injuries directly causing by HBV in situ were most involved in the pathogenesis of IgAN. While in another way, considering the prevalence of HBV infection could reach 5–7.99% of the population in China, therefore, whether IgAN was secondary to HBV infection, or presenting as a geographical coincidence, both of them should be further carefully demonstrated. Besides, previous research of IgAN has revealed that in vitro EBV-transformed peripheral-blood cells from healthy individuals produce almost exclusively IgA1 subclass. In a recent study of Zachova et al. (2020), which indicates EBV infection may be involved in the pathogenesis of IgAN. They found that B cells and their IgA^+^ subpopulation in peripheral blood of IgAN patients displayed a significantly higher frequency of EBV infection compared to the controls and displayed increased expression of homing receptors for targeting the upper respiratory tract. Similarly, in the animal model of IgAN, by oral immunization with the Sendai virus, a parainfluenza virus likewise to human respiratory tract viruses can induce IgAN in mice (Suzuki & Suzuki, 2018). These in vivo and in vitro phenotypes had a raised speculation, that different types of viruses infection have shared some similar molecular biological processes, which aroused response of circRNAs expression and involved in the disease progression of IgAN.

Moreover, the GO enrichment analysis has shown that the circRNAs-associated genes’ most related cellular component were intracellular membrane-bounded organelle and cytoplasm, especially the process associated with vesicle transport between the ER to Golgi. Although the molecule function of intracellular vesicle transport is relatively less discuss in IgAN, however, it’s naturally closely related to the immunoglobulin’s processing (Kirk et al., 2010). For instance, the C1GALT1, which function as a crucial enzyme of process IgA1 galactosylation, normally C1GALT1 is synthesized in ER under the help of molecular chaperone Cosmc, and it’s necessary to be packaged in vesicles and transport to Golgi then finish the galactosylation of IgA1 in Golgi (Cummings, 2019). In IgAN, the significant deficiency of C1GALT1 (expression or/and activity) have resulted in the elevated of Gd-IgA1, and directly correlated with serum levels of Gd-IgA1 in IgAN patients which have been demonstrated by many works of literature (Lai et al., 2016). In our previous study, Wang et al. (2019) have illustrated the Golgi matrix protein 130 plays the role of docking C1GALT1 vesicles in Golgi, the dysregulation with this molecular process had been demonstrated associated with Gd-IgA1 in IgAN, and firstly aroused the attention about the molecular process of ER-Golgi transportation in IgAN. Combined with our findings according to the ceRNA network, exploring the virus infection-related intracellular vesicle transport mechanism might be an interesting direction of IgAN in the future.

Lastly, our study was designed as combined with the patient’s data from Italian, Japanese and Chinese populations, for better avoiding the bias brought by single-center sample, but in another way, the different race’ sample sources bring the heterogeneity challenges to the comprehensive analysis of genes profile and validation of hub genes. For example, the molecular in this ceRNA network was relatively less than other ceRNA comprehensively studies, and we couldn’t find ceRNA interaction pair with certain order (up-down-up or down-up-down) to validates the potential associated relationship corresponding with GALNT, ASB16 or SEC24C. The small sample size of the hub gene verification is also the one of limitations of this study, validation of a larger sample size will be needed, and exploring the potential capability of circRNAs as biomarkers of IgAN would be a research in the future. Future research should further identify the hub molecules with therapeutic potential in IgAN and conducted the overexpression or inhibition experiments of related ceRNAs, and observing the reciprocal relationship of target genes, for a better understanding of the molecular pathogenesis of IgAN.

## Conclusions

In summary, we successfully identified IgAN associated circRNAs using RNA-seq analysis, and elucidated the circRNA-associated ceRNA networks of IgAN through the integrated analysis of the RNA expression profile. To our knowledge, this is the first report examining the expression of circRNAs in IgAN. These findings have expanded our understanding of circRNAs-associated ceRNA networks in IgAN. In the future, exploring the related molecular regulatory mechanism will be needed for a better understanding of IgAN.

##  Supplemental Information

10.7717/peerj.10395/supp-1 Supplemental Information 1 Top 10 processes revealed in GO enrichment analysis for the DEGs Top 10 GO enrichment process of (A) up-regulated DEGs and (B) down-regulated DEGs, categorized by biological process (BP), molecular function (MF), and cellular component (CC). Red indicates higher enrichment, the sizes of the dots represent the numbers of genes in each GO category.

10.7717/peerj.10395/supp-2 Supplemental Information 2 Primer sequences for the hub genes in the ceRNA network

10.7717/peerj.10395/supp-3 Supplemental Information 3 Dysregulated circRNA transcripts between IgAN patients and healthy controls

10.7717/peerj.10395/supp-4 Supplemental Information 4 Dysregulated miRNA transcripts between controls and IgAN patients

10.7717/peerj.10395/supp-5 Supplemental Information 5 Dysregulated mRNA transcripts between IgAN patients and controls

10.7717/peerj.10395/supp-6 Supplemental Information 6 Markedly enriched GO terms and KEGG pathways of the DEGs bewteen IgAN patients and controls

10.7717/peerj.10395/supp-7 Supplemental Information 7 CircRNA-miRNA-mRNA ceRNA network

10.7717/peerj.10395/supp-8 Supplemental Information 8 Markedly enriched GO terms and KEGG pathways of the genes in the circRNA-associated-ceRNA networks

10.7717/peerj.10395/supp-9 Supplemental Information 9 Raw data for qRT-PCR

## Additional Information and Declarations

### Data Availability

The following information was supplied regarding data availability: The raw data are available in the Supplemental File and the circRNA sequences are available at GEO: GSE154046.
