# Supplementary material for: Comprehensive analysis of circRNA expression profiles and circRNA-associated competing endogenous RNA networks in IgA nephropathy
Source: PeerJ. 2020 Dec 3;8:e10395. doi: 10.7717/peerj.10395 (PMC7719294; doi:10.7717/peerj.10395)
Supplement: Supplemental Information 1 — Top 10 GO enrichment process of (A) up-regulated DEGs and (B) down-regulated DEGs, categorized by biological process (BP), molecular function (MF), and cellular component (CC). Red indicates higher enrichment, the sizes of the dots represent the numbers of genes in each GO category. [file peerj-08-10395-s001.pdf]

A

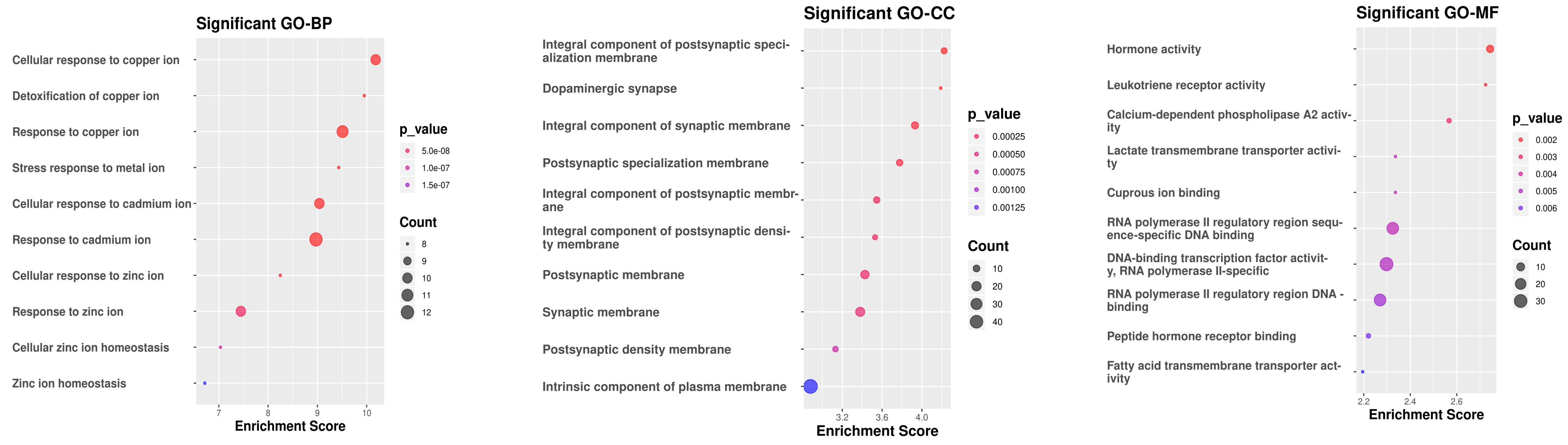
Significant GO-BP
Cellular response to copper ion
Detoxification of copper ion
Response to copper ion
Stress response to metal ion
Cellular response to cadmium ion
Response to cadmium ion
Cellular response to zinc ion
Response to zinc ion
Cellular zinc ion homeostasis
Zinc ion homeostasis
7
8
9
10
Enrichment Score
p_value
5.0e-08
1.0e-07
1.5e-07
Count
8
9
10
11
12
Significant GO-CC
Integral component of postsynaptic speci-
alization membrane
Dopaminergic synapse
Integral component of synaptic membrane
Postsynaptic specialization membrane
Integral component of postsynaptic membr-
ane
Integral component of postsynaptic densi-
ty membrane
Postsynaptic membrane
Synaptic membrane
Postsynaptic density membrane
Intrinsic component of plasma membrane
3.2
3.6
4.0
Enrichment Score
p_value
0.00025
0.00050
0.00075
0.00100
0.00125
Count
10
20
30
40
Significant GO-MF
Hormone activity
Leukotriene receptor activity
Calcium-dependent phospholipase A2 activ-
ity
Lactate transmembrane transporter activi-
ty
Cuprous ion binding
RNA polymerase II regulatory region sequ-
ence-specific DNA binding
DNA-binding transcription factor activit-
y, RNA polymerase II-specific
RNA polymerase II regulatory region DNA -
binding
Peptide hormone receptor binding
Fatty acid transmembrane transporter act-
ivity
2.2
2.4
2.6
Enrichment Score
p_value
0.002
0.003
0.004
0.005
0.006
Count
10
20
30

B

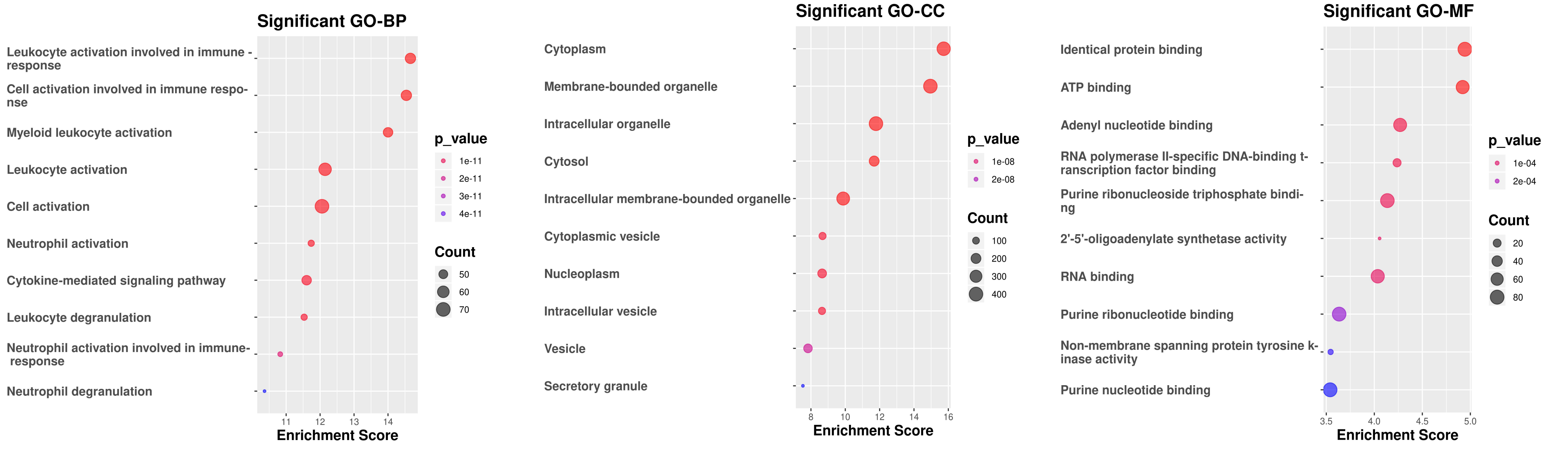
Significant GO-BP
Leukocyte activation involved in immune -
response
Cell activation involved in immune respo-
nse
Myeloid leukocyte activation
Leukocyte activation
Cell activation
Neutrophil activation
Cytokine-mediated signaling pathway
Leukocyte degranulation
Neutrophil activation involved in immune-
response
Neutrophil degranulation
11
12
13
14
Enrichment Score
p_value
1e-11
2e-11
3e-11
4e-11
Count
50
60
70
Significant GO-CC
Cytoplasm
Membrane-bounded organelle
Intracellular organelle
Cytosol
Intracellular membrane-bounded organelle
Cytoplasmic vesicle
Nucleoplasm
Intracellular vesicle
Vesicle
Secretory granule
8
10
12
14
16
Enrichment Score
p_value
1e-08
2e-08
Count
100
200
300
400
Significant GO-MF
Identical protein binding
ATP binding
Adenyl nucleotide binding
RNA polymerase II-specific DNA-binding t-
ranscription factor binding
Purine ribonucleoside triphosphate bindi-
ng
2'-5'-oligoadenylate synthetase activity
RNA binding
Purine ribonucleotide binding
Non-membrane spanning protein tyrosine k-
inase activity
Purine nucleotide binding
3.5
4.0
4.5
5.0
Enrichment Score
p_value
1e-04
2e-04
Count
20
40
60
80

Figure S1 Top 10 processes revealed in GO enrichment analysis for the DEGs.

Top 10 GO enrichment process of (A) up-regulated DEGs and (B) down-regulated DEGs, categorized by biological process (BP), molecular function (MF), and cellular component (CC). Red indicates higher enrichment, the sizes of the dots represent the numbers of genes in each GO category.
