# Supplementary material for: Comprehensive analysis of circRNA expression profiles and circRNA-associated competing endogenous RNA networks in IgA nephropathy
Source: PeerJ. 2020 Dec 3;8:e10395. doi: 10.7717/peerj.10395 (PMC7719294; doi:10.7717/peerj.10395)
Supplement: Supplemental Information 2 [file peerj-08-10395-s002.docx]

**Table S1.** **Primer sequences for the hub genes in the ceRNA network.**

|  | **Forward** | **Reverse** |
| --- | --- | --- |
| H-ASB16 | GACTGTGCTCGACACCTGAT | CAGAGGTGCAAAGGAGTCGT |
| H-TRIM21 | CCCCTCTAACCCTCTGTCCA | CTGCTAAAGCTCGCTTGCTG |
| H-HLA-B | CTAGCAGTTGTGGTCATCGGA | AAAGGGGAGGCGTGAAGAAA |
| H-SEC24C | CCTGGGAGCCTTGACGTTAG | CCCCAGCCAGCATTCAGATT |
| H-β-Actin* | ACCCTGAAGTACCCCATCGAG | AGCACAGCCTGGATAGCAAC |

*β-Actin was used as the house-keeping gene for normalization.
