# Supplementary material for: Comprehensive analysis of circRNA expression profiles and circRNA-associated competing endogenous RNA networks in IgA nephropathy
Source: PeerJ. 2020 Dec 3;8:e10395. doi: 10.7717/peerj.10395 (PMC7719294; doi:10.7717/peerj.10395)
Supplement: Supplemental Information 3 [file peerj-08-10395-s003.docx]

**Table S2. Dysregulated circRNA transcripts between IgAN patients and healthy controls.**

| **Accession** | **CircBase** ID | **Gene symbol** | **Ctrl1_**  **Ctrl** | **Ctrl2_**  **Ctrl** | **Ctrl3_**  **Ctrl** | **IgAN1_**  **IgAN** | **IgAN2_**  **IgAN** | **IgAN3_**  **IgAN** | **IgAN** | **Ctrl** | **log_2_(FC)** | **P-value** |
| --- | --- | --- | --- | --- | --- | --- | --- | --- | --- | --- | --- | --- |
| circRNA10024 | novel_circ | RPS6KA5 | 0 | 0 | 0 | 54.197408 | 38.811309 | 49.328375 | 47.445698 | 0 | 5.598297 | 0.009033 |
| circRNA10560 | hsa_circ_0062003 | ADARB1 | 0 | 0 | 0 | 5.772147 | 5.267758 | 4.821299 | 5.287068 | 0 | 2.652387 | 0.002688 |
| circRNA10571 | novel_circ | RNF216P1 | 0 | 0 | 0 | 14.430856 | 13.047326 | 16.169426 | 14.549203 | 0 | 3.958769 | 0.003832 |
| circRNA10637 | novel_circ | SEPT7-AS1 | 0 | 0 | 0 | 9.182331 | 7.532801 | 5.602373 | 7.439169 | 0 | 3.077101 | 0.018795 |
| circRNA10666 | novel_circ | SBDSP1 | 0 | 0 | 0 | 11.771466 | 17.113515 | 18.928244 | 15.937742 | 0 | 4.08217 | 0.017684 |
| circRNA10718 | novel_circ | CCDC132 | 0 | 0 | 0 | 6.062825 | 7.462521 | 5.931407 | 6.485584 | 0 | 2.904115 | 0.005658 |
| circRNA10745 | hsa_circ_0007612 | ORC5 | 0 | 0 | 0 | 30.711038 | 25.79246 | 48.057979 | 34.853826 | 0 | 5.164055 | 0.03555 |
| circRNA10752 | hsa_circ_0081828 | SRPK2 | 0 | 0 | 0 | 5.637752 | 6.789124 | 6.142621 | 6.189832 | 0 | 2.845958 | 0.002885 |
| circRNA10812 | hsa_circ_0082689 | PARP12 | 0 | 0 | 0 | 6.900935 | 6.3039 | 8.328839 | 7.177891 | 0 | 3.031729 | 0.006932 |
| circRNA10989 | hsa_circ_0009144 | SEC63 | 0 | 0 | 0 | 7.934319 | 9.331105 | 8.063379 | 8.442934 | 0 | 3.239235 | 0.002774 |
| circRNA11010 | hsa_circ_0077735 | CEP85L | 0 | 0 | 0 | 16.213696 | 13.698156 | 24.144923 | 18.018925 | 0 | 4.249364 | 0.02919 |
| circRNA11022 | novel_circ | SAMD3 | 0 | 0 | 0 | 14.469801 | 13.950733 | 26.167917 | 18.196151 | 0 | 4.262745 | 0.044844 |
| circRNA11045 | hsa_circ_0009096 | UTRN | 0 | 0 | 0 | 19.364182 | 15.708681 | 29.670865 | 21.581242 | 0 | 4.497053 | 0.035531 |
| circRNA11137 | novel_circ | EMB | 0 | 0 | 0 | 58.35642 | 60.217256 | 71.966725 | 63.513467 | 0 | 6.011528 | 0.00447 |
| circRNA11286 | novel_circ | HSPA4 | 0 | 0 | 0 | 9.330029 | 12.869194 | 10.909619 | 11.036281 | 0 | 3.589318 | 0.008493 |
| circRNA11327 | novel_circ | FAM114A2 | 0 | 0 | 0 | 6.337994 | 5.986567 | 5.426267 | 5.916943 | 0 | 2.790135 | 0.002007 |
| circRNA11402 | hsa_circ_0069285 | LCORL | 0 | 0 | 0 | 6.535861 | 6.113957 | 5.875222 | 6.175013 | 0 | 2.842982 | 0.000977 |
| circRNA11518 | novel_circ | HERC3 | 0 | 0 | 0 | 36.190744 | 31.735852 | 34.275768 | 34.067455 | 0 | 5.132061 | 0.001431 |
| circRNA11571 | hsa_circ_0001438 | LARP1B | 0 | 0 | 0 | 2.892252 | 3.827929 | 4.840146 | 3.853442 | 0 | 2.279008 | 0.020647 |
| circRNA11578 | hsa_circ_0070960 | SCLT1 | 0 | 0 | 0 | 4.77893 | 3.354849 | 3.625362 | 3.919714 | 0 | 2.298574 | 0.012183 |
| circRNA11784 | hsa_circ_0006429 | SUCLG2 | 0 | 0 | 0 | 10.440097 | 9.169272 | 13.64391 | 11.084426 | 0 | 3.595077 | 0.01412 |
| circRNA11806 | hsa_circ_0066719 | CBLB | 0 | 0 | 0 | 11.158576 | 9.766615 | 14.625495 | 11.850228 | 0 | 3.683722 | 0.014538 |
| circRNA11927 | hsa_circ_0002832 | GMPS | 0 | 0 | 0 | 3.435489 | 4.661665 | 3.858412 | 3.985189 | 0 | 2.317648 | 0.008044 |
| circRNA12056 | novel_circ | ROCK2 | 0 | 0 | 0 | 10.53237 | 10.258565 | 11.535014 | 10.775317 | 0 | 3.557694 | 0.001294 |
| circRNA12115 | hsa_circ_0003288 | BIRC6 | 0 | 0 | 0 | 4.64596 | 4.14994 | 4.637966 | 4.477955 | 0 | 2.453638 | 0.001339 |
| circRNA12195 | hsa_circ_0008534 | PEX13 | 0 | 0 | 0 | 4.649243 | 3.632424 | 4.23782 | 4.173162 | 0 | 2.371046 | 0.00497 |
| circRNA12229 | hsa_circ_0055019 | APLF | 0 | 0 | 0 | 3.829538 | 4.382716 | 4.631319 | 4.281191 | 0 | 2.400863 | 0.003049 |
| circRNA12492 | novel_circ | SP140L | 0 | 0 | 0 | 61.120606 | 61.991707 | 63.677822 | 62.263378 | 0 | 5.983299 | 0.000145 |
| circRNA12528 | novel_circ | EXOSC10 | 0 | 0 | 0 | 17.977344 | 21.288603 | 17.643553 | 18.969833 | 0 | 4.31975 | 0.00374 |
| circRNA12604 | novel_circ | AGO4 | 0 | 0 | 0 | 8.026462 | 7.894859 | 5.578477 | 7.166599 | 0 | 3.029735 | 0.012082 |
| circRNA12647 | hsa_circ_0012545 | ZCCHC11 | 0 | 0 | 0 | 16.694785 | 12.770639 | 17.311985 | 15.59247 | 0 | 4.052457 | 0.008216 |
| circRNA12705 | novel_circ | CCDC18 | 0 | 0 | 0 | 2.367423 | 2.327582 | 2.697979 | 2.464328 | 0 | 1.792575 | 0.002261 |
| circRNA12818 | hsa_circ_0004680 | CCT3 | 0 | 0 | 0 | 17.156126 | 18.513674 | 17.252322 | 17.640707 | 0 | 4.220385 | 0.000614 |
| circRNA12995 | novel_circ | TBCE | 0 | 0 | 0 | 2.84013 | 1.8702 | 3.352186 | 2.687505 | 0 | 1.882645 | 0.025164 |
| circRNA1357 | hsa_circ_0006845 | NFAT5 | 2.678158 | 3.755849 | 4.18247 | 6.37465 | 8.386073 | 10.477218 | 8.412647 | 3.538826 | 1.052281 | 0.040813 |
| circRNA1382 | hsa_circ_0004087 | CDYL2 | 2.207671 | 1.942943 | 0 | 4.703036 | 5.229522 | 3.186328 | 4.372962 | 1.383538 | 1.172613 | 0.03289 |
| circRNA1386 | hsa_circ_0040719 | ZDHHC7 | 15.328736 | 18.922578 | 0 | 41.148148 | 41.417212 | 34.285633 | 38.950331 | 11.417105 | 1.685879 | 0.028407 |
| circRNA154 | hsa_circ_0030051 | ELF1 | 22.909604 | 26.595583 | 25.708843 | 47.086022 | 63.428637 | 49.434794 | 53.316485 | 25.071344 | 1.058925 | 0.026595 |
| circRNA1587 | hsa_circ_0007201 | IQGAP1 | 10.401064 | 0 | 0 | 23.323762 | 26.912405 | 24.050802 | 24.762323 | 3.467021 | 2.527878 | 0.018047 |
| circRNA1622 | hsa_circ_0002928 | PRMT5 | 3.896378 | 3.896857 | 0 | 7.493562 | 8.599178 | 7.879307 | 7.990682 | 2.597745 | 1.321338 | 0.046291 |
| circRNA1636 | hsa_circ_0031583 | ARHGAP5 | 3.482024 | 3.727662 | 0 | 6.390574 | 7.632487 | 9.767501 | 7.930187 | 2.403229 | 1.391786 | 0.025305 |
| circRNA1639 | novel_circ | SNX6 | 12.229394 | 12.807355 | 19.620421 | 26.121974 | 37.808607 | 29.326397 | 31.085659 | 14.885723 | 1.014198 | 0.023276 |
| circRNA178 | hsa_circ_0000483 | SETDB2 | 3.717663 | 0 | 0 | 4.769149 | 5.028694 | 8.025839 | 5.941228 | 1.239221 | 1.632194 | 0.045611 |
| circRNA1902 | novel_circ | TGFB1 | 18.724198 | 0 | 0 | 58.058816 | 61.167079 | 27.628961 | 48.951619 | 6.241399 | 2.786191 | 0.036735 |
| circRNA2075 | hsa_circ_0008870 | MAPK1 | 13.916291 | 13.977234 | 15.509656 | 26.847078 | 35.164052 | 30.165283 | 30.725471 | 14.467727 | 1.03638 | 0.017766 |
| circRNA2136 | hsa_circ_0002077 | NUP50 | 14.679023 | 13.784717 | 11.88159 | 27.11772 | 36.133492 | 35.039742 | 32.763652 | 13.448443 | 1.224557 | 0.015096 |
| circRNA2295 | hsa_circ_0001185 | IFNGR2 | 6.635257 | 0 | 0 | 16.733709 | 22.835855 | 18.341689 | 19.303751 | 2.211752 | 2.660314 | 0.004449 |
| circRNA2305 | hsa_circ_0001187 | DOPEY2 | 3.031206 | 3.738264 | 3.923529 | 10.129608 | 11.344735 | 8.190815 | 9.888386 | 3.564333 | 1.254314 | 0.014504 |
| circRNA2349 | hsa_circ_0007177 | CCZ1 | 5.842615 | 4.748305 | 5.091469 | 12.837367 | 11.959865 | 12.433931 | 12.410388 | 5.227463 | 1.106634 | 0.000092 |
| circRNA2512 | hsa_circ_0082096 | ZNF800 | 11.716505 | 0 | 0 | 17.641478 | 23.471092 | 25.292704 | 22.135091 | 3.905502 | 2.23761 | 0.023863 |
| circRNA2633 | hsa_circ_0006757 | C6orf106 | 8.619002 | 8.727878 | 0 | 16.890752 | 21.145351 | 17.019303 | 18.351802 | 5.782293 | 1.512623 | 0.031787 |
| circRNA2922 | hsa_circ_0002490 | FCHO2 | 2.898384 | 1.959334 | 3.768005 | 6.292807 | 7.998657 | 6.297566 | 6.86301 | 2.875241 | 1.020795 | 0.006783 |
| circRNA2950 | hsa_circ_0073237 | VCAN | 9.769469 | 11.839281 | 21.139619 | 45.078967 | 51.943749 | 37.259942 | 44.760886 | 14.249457 | 1.585357 | 0.005725 |
| circRNA2980 | hsa_circ_0002919 | AP3S1 | 12.329635 | 0 | 0 | 18.935744 | 27.876699 | 36.175289 | 27.662577 | 4.109878 | 2.487807 | 0.023163 |
| circRNA3024 | hsa_circ_0001538 | PAIP2 | 33.906497 | 34.236272 | 0 | 74.391413 | 93.395424 | 70.80382 | 79.530219 | 22.714256 | 1.763776 | 0.019224 |
| circRNA3087 | hsa_circ_0002051 | CANX | 29.577344 | 26.35098 | 37.064358 | 64.086453 | 70.22021 | 70.027643 | 68.111435 | 30.997561 | 1.110963 | 0.001301 |
| circRNA3195 | hsa_circ_0007883 | ANKRD17 | 5.396476 | 6.315991 | 4.213978 | 10.573695 | 15.524839 | 10.219567 | 12.106034 | 5.308815 | 1.05479 | 0.04537 |
| circRNA3200 | hsa_circ_0007096 | USO1 | 4.800473 | 0 | 0 | 6.870284 | 7.133608 | 10.850193 | 8.284695 | 1.600158 | 1.836255 | 0.033301 |
| circRNA3228 | hsa_circ_0002538 | KLHL8 | 2.777248 | 2.180258 | 3.404482 | 7.993491 | 9.606241 | 10.159368 | 9.253034 | 2.787329 | 1.436798 | 0.002814 |
| circRNA3239 | hsa_circ_0070562 | TET2 | 6.515139 | 7.658741 | 8.952916 | 21.396911 | 26.770182 | 18.811817 | 22.326303 | 7.708932 | 1.42139 | 0.017967 |
| circRNA3302 | novel_circ | FBXW7 | 10.032744 | 8.953344 | 8.523336 | 21.734808 | 23.538036 | 18.734959 | 21.335934 | 9.169808 | 1.135074 | 0.008017 |
| circRNA332 | hsa_circ_0007889 | EIF4B | 39.201541 | 41.073648 | 0 | 88.977909 | 104.768757 | 89.796826 | 94.514498 | 26.758396 | 1.782796 | 0.025098 |
| circRNA3365 | novel_circ | RAB5A | 9.568311 | 5.111666 | 0 | 14.543735 | 16.020553 | 19.175032 | 16.579773 | 4.893325 | 1.576763 | 0.033734 |
| circRNA3627 | hsa_circ_0002004 | FXR1 | 10.588645 | 0 | 0 | 23.479463 | 24.021218 | 23.249428 | 23.58337 | 3.529548 | 2.440244 | 0.029169 |
| circRNA3757 | hsa_circ_0000992 | PRKD3 | 7.583445 | 8.989013 | 9.828361 | 20.172768 | 23.380279 | 15.160355 | 19.571134 | 8.800273 | 1.069728 | 0.038063 |
| circRNA3763 | hsa_circ_0000994 | SLC8A1 | 2.799743 | 3.100581 | 0 | 7.512277 | 5.867469 | 6.999767 | 6.793171 | 1.966775 | 1.393315 | 0.023279 |
| circRNA4065 | hsa_circ_0058218 | ARPC2 | 64.099861 | 0 | 0 | 135.90721 | 164.699906 | 97.17682 | 132.594646 | 21.36662 | 2.578443 | 0.018734 |
| circRNA4136 | hsa_circ_0000014 | LZIC | 2.418448 | 1.732327 | 1.660592 | 5.433466 | 5.93679 | 5.107327 | 5.492528 | 1.937123 | 1.144377 | 0.00048 |
| circRNA4186 | hsa_circ_0005576 | CDC42 | 114.142672 | 107.359069 | 130.012156 | 229.485361 | 280.713186 | 240.233604 | 250.14405 | 117.171299 | 1.087635 | 0.006124 |
| circRNA4197 | hsa_circ_0000032 | TMEM50A | 13.455629 | 0 | 0 | 26.938885 | 27.96949 | 20.475405 | 25.127927 | 4.48521 | 2.251974 | 0.026322 |
| circRNA4241 | hsa_circ_0000048 | RNF19B | 5.374456 | 5.164816 | 0 | 11.512976 | 10.182933 | 14.425193 | 12.040367 | 3.513091 | 1.530797 | 0.02041 |
| circRNA4243 | hsa_circ_0009027 | ZNF362 | 6.009402 | 3.221912 | 0 | 10.752372 | 11.202786 | 7.091376 | 9.682178 | 3.077105 | 1.389589 | 0.042244 |
| circRNA4417 | hsa_circ_0000116 | MAN1A2 | 7.772011 | 8.629755 | 0 | 15.15581 | 22.939072 | 18.639807 | 18.911563 | 5.467255 | 1.622381 | 0.020682 |
| circRNA4468 | hsa_circ_0008368 | TPM3 | 19.249018 | 0 | 18.609913 | 47.708386 | 60.479564 | 36.945195 | 48.377715 | 12.619643 | 1.858171 | 0.018441 |
| circRNA4472 | hsa_circ_0014528 | CLK2 | 19.341527 | 20.900988 | 0 | 35.607818 | 51.018118 | 40.67166 | 42.432532 | 13.414172 | 1.591288 | 0.02888 |
| circRNA4486 | hsa_circ_0014614 | DAP3 | 4.196349 | 2.953679 | 0 | 6.284205 | 7.663061 | 8.846668 | 7.597978 | 2.383343 | 1.345548 | 0.032074 |
| circRNA4493 | hsa_circ_0008339 | KIAA0907 | 14.091012 | 12.790492 | 0 | 31.572197 | 27.676199 | 20.587867 | 26.612087 | 8.960501 | 1.47101 | 0.038088 |
| circRNA4584 | novel_circ | ELK4 | 10.935702 | 11.729477 | 7.822666 | 19.748345 | 24.743952 | 20.261124 | 21.584474 | 10.162615 | 1.016656 | 0.005664 |
| circRNA4866 | hsa_circ_0089371 | REXO4 | 3.137126 | 3.550983 | 0 | 8.805111 | 7.862145 | 5.874513 | 7.513923 | 2.22937 | 1.398571 | 0.022627 |
| circRNA4937 | hsa_circ_0001802 | PCMTD1 | 8.795069 | 0 | 0 | 15.24052 | 16.126868 | 13.974543 | 15.113977 | 2.93169 | 2.035091 | 0.047914 |
| circRNA512 | hsa_circ_0005092 | IPO7 | 7.163943 | 7.616415 | 0 | 12.657093 | 16.585872 | 18.600599 | 15.947855 | 4.926786 | 1.515781 | 0.026145 |
| circRNA5193 | hsa_circ_0025887 | LRRK2 | 0 | 10.856192 | 0 | 25.937634 | 24.115241 | 23.907107 | 24.653327 | 3.618731 | 2.473578 | 0.025334 |
| circRNA534 | hsa_circ_0000284 | HIPK3 | 13.647863 | 13.412512 | 16.753593 | 34.217789 | 36.296508 | 27.040577 | 32.518292 | 14.604656 | 1.102972 | 0.014305 |
| circRNA5376 | novel_circ | ARHGAP21 | 0 | 2.423376 | 0 | 5.886434 | 5.010438 | 2.988583 | 4.628485 | 0.807792 | 1.638518 | 0.031778 |
| circRNA562 | hsa_circ_0004162 | PATL1 | 11.876037 | 12.126091 | 13.137291 | 23.401124 | 32.706522 | 30.436788 | 28.848145 | 12.379806 | 1.157584 | 0.025895 |
| circRNA5877 | hsa_circ_0004658 | EMILIN2 | 0 | 4.629768 | 5.671904 | 13.797634 | 16.462757 | 10.134687 | 13.465026 | 3.43389 | 1.705924 | 0.016703 |
| circRNA6006 | novel_circ | MCM3AP | 0 | 8.986113 | 0 | 14.967404 | 15.185171 | 17.580704 | 15.911093 | 2.995371 | 2.081569 | 0.041414 |
| circRNA6047 | hsa_circ_0005878 | SBDSP1 | 0 | 6.701923 | 0 | 9.749156 | 12.749117 | 18.655927 | 13.718067 | 2.233974 | 2.186208 | 0.029969 |
| circRNA6172 | novel_circ | CNPY3 | 0 | 34.779453 | 0 | 76.473773 | 69.998462 | 92.510228 | 79.660821 | 11.593151 | 2.679229 | 0.012547 |
| circRNA6178 | hsa_circ_0004092 | MB21D1 | 0 | 1.529832 | 0 | 3.229071 | 3.13955 | 1.675599 | 2.681407 | 0.509944 | 1.285762 | 0.038792 |
| circRNA6296 | hsa_circ_0007559 | HEXB | 0 | 12.168087 | 15.231683 | 30.731257 | 43.926507 | 40.748954 | 38.468906 | 9.133257 | 1.961619 | 0.009217 |
| circRNA6408 | hsa_circ_0070040 | NUP54 | 0 | 2.028901 | 0 | 4.097103 | 5.174946 | 5.225225 | 4.832425 | 0.6763 | 1.798815 | 0.011554 |
| circRNA6572 | hsa_circ_0006840 | TRA2B | 0 | 37.632277 | 0 | 48.549343 | 77.363701 | 88.027618 | 71.313554 | 12.544092 | 2.416602 | 0.027097 |
| circRNA6660 | hsa_circ_0008609 | C2orf15 | 0 | 2.150622 | 0 | 4.439071 | 5.336682 | 2.941608 | 4.23912 | 0.716874 | 1.609541 | 0.024502 |
| circRNA6686 | hsa_circ_0001072 | GTDC1 | 0 | 3.496307 | 0 | 9.402527 | 8.791664 | 8.717406 | 8.970532 | 1.165436 | 2.203013 | 0.018761 |
| circRNA6779 | hsa_circ_0011162 | TAF12 | 0 | 3.070429 | 0 | 4.073693 | 6.549564 | 5.983498 | 5.535585 | 1.023476 | 1.69148 | 0.027331 |
| circRNA7634 | novel_circ | NIPBL | 0 | 0 | 6.51887 | 8.41656 | 10.70725 | 13.030658 | 10.718156 | 2.172957 | 1.884846 | 0.03784 |
| circRNA8160 | hsa_circ_0000467 | SKA3 | 0 | 0 | 0 | 3.184285 | 4.160258 | 3.012114 | 3.452219 | 0 | 2.154525 | 0.010554 |
| circRNA846 | hsa_circ_0002681 | CCAR1 | 3.453904 | 3.488731 | 3.629417 | 6.942912 | 9.403347 | 8.501439 | 8.282566 | 3.524018 | 1.036919 | 0.021557 |
| circRNA8550 | hsa_circ_0028670 | TAOK3 | 0 | 0 | 0 | 39.833783 | 38.19896 | 51.890044 | 43.307596 | 0 | 5.469482 | 0.009791 |
| circRNA8583 | hsa_circ_0029309 | NCOR2 | 0 | 0 | 0 | 11.561637 | 11.929105 | 15.641074 | 13.043939 | 0 | 3.811876 | 0.00983 |
| circRNA8855 | hsa_circ_0004277 | WDR37 | 0 | 0 | 0 | 6.5214 | 6.01119 | 8.856145 | 7.129578 | 0 | 3.023181 | 0.014755 |
| circRNA8857 | hsa_circ_0017510 | PITRM1 | 0 | 0 | 0 | 7.135898 | 7.067157 | 5.131827 | 6.444961 | 0 | 2.896264 | 0.010228 |
| circRNA8946 | novel_circ | BMS1P1 | 0 | 0 | 0 | 1.715035 | 1.53935 | 2.469874 | 1.908087 | 0 | 1.54007 | 0.021654 |
| circRNA8965 | hsa_circ_0018478 | HERC4 | 0 | 0 | 0 | 8.987419 | 5.429639 | 10.156063 | 8.191041 | 0 | 3.200228 | 0.028814 |
| circRNA9138 | hsa_circ_0042098 | MAP2K4 | 0 | 0 | 0 | 7.764319 | 7.279467 | 8.614709 | 7.886165 | 0 | 3.151561 | 0.00244 |
| circRNA92 | novel_circ | MAP7D3 | 1.355385 | 0 | 0 | 3.230707 | 4.735269 | 1.994973 | 3.320317 | 0.451795 | 1.573299 | 0.047611 |
| circRNA9340 | hsa_circ_0008438 | TLK2 | 0 | 0 | 0 | 7.250886 | 8.382308 | 5.419629 | 7.017608 | 0 | 3.003172 | 0.014794 |
| circRNA9482 | hsa_circ_0038725 | IL4R | 0 | 0 | 0 | 67.101662 | 74.210253 | 51.905176 | 64.405697 | 0 | 6.031344 | 0.010272 |
| circRNA9561 | hsa_circ_0040210 | DDX19B | 0 | 0 | 0 | 9.027011 | 10.361441 | 7.286552 | 8.891668 | 0 | 3.306214 | 0.009875 |
| circRNA9751 | hsa_circ_0035801 | HERC1 | 0 | 0 | 0 | 6.045008 | 4.417637 | 4.84458 | 5.102408 | 0 | 2.609379 | 0.008993 |
| circRNA9772 | hsa_circ_0008798 | MAP2K1 | 0 | 0 | 0 | 18.390845 | 18.483068 | 10.722901 | 15.865605 | 0 | 4.076012 | 0.025278 |
| circRNA1025 | hsa_circ_0000754 | SSH2 | 42.997056 | 59.021293 | 61.466487 | 24.708181 | 19.509053 | 29.829269 | 24.682168 | 54.494945 | -1.111589 | 0.01973 |
| circRNA1147 | hsa_circ_0006690 | CCDC47 | 2.911769 | 2.518155 | 3.651447 | 0 | 0 | 0 | 0 | 3.027124 | -2.00975 | 0.01183 |
| circRNA1416 | novel_circ | FMN1 | 7.675839 | 16.635766 | 14.356504 | 4.985212 | 4.347409 | 0 | 3.110874 | 12.88937 | -1.756464 | 0.046869 |
| circRNA188 | hsa_circ_0005783 | KLF12 | 14.581428 | 15.588023 | 9.416365 | 0 | 0 | 0 | 0 | 13.195272 | -3.827339 | 0.02035 |
| circRNA2314 | hsa_circ_0005955 | DYRK1A | 125.2663 | 143.375151 | 138.792791 | 71.504033 | 47.960056 | 0 | 39.821363 | 135.811414 | -1.744792 | 0.037735 |
| circRNA2390 | hsa_circ_0006773 | HIBADH | 29.804462 | 34.421415 | 27.600677 | 16.351159 | 12.982742 | 0 | 9.777967 | 30.608851 | -1.552244 | 0.038265 |
| circRNA2400 | hsa_circ_0003162 | BBS9 | 15.358969 | 17.415067 | 15.727162 | 6.891605 | 3.197099 | 0 | 3.362901 | 16.167066 | -1.976284 | 0.016191 |
| circRNA2569 | hsa_circ_0008206 | KMT2C | 12.601776 | 14.044168 | 13.289713 | 0 | 0 | 0 | 0 | 13.311886 | -3.839142 | 0.000978 |
| circRNA259 | novel_circ | ITPR2 | 2.662896 | 2.936087 | 3.354936 | 0 | 0 | 0 | 0 | 2.98464 | -1.994449 | 0.004516 |
| circRNA2599 | hsa_circ_0075533 | FARS2 | 41.88698 | 40.204692 | 39.345562 | 16.390749 | 8.736674 | 0 | 8.375808 | 40.479078 | -2.145369 | 0.019022 |
| circRNA27 | hsa_circ_0006364 | ZNF81 | 31.491009 | 28.897634 | 34.0466 | 14.604558 | 13.407816 | 9.36384 | 12.458738 | 31.478414 | -1.270938 | 0.000957 |
| circRNA2846 | novel_circ | GUSBP1 | 51.412404 | 36.375284 | 38.610308 | 18.714291 | 14.017472 | 25.378228 | 19.369997 | 42.132665 | -1.082335 | 0.020315 |
| circRNA3165 | hsa_circ_0002021 | FRYL | 98.82969 | 109.501373 | 126.545926 | 39.502627 | 28.371964 | 0 | 22.624863 | 111.625663 | -2.253158 | 0.004991 |
| circRNA3482 | hsa_circ_0066536 | EIF4E3 | 68.886057 | 61.591925 | 63.474771 | 31.310164 | 25.914597 | 0 | 19.074921 | 64.650918 | -1.709421 | 0.036614 |
| circRNA3506 | novel_circ | CD96 | 265.581905 | 325.699856 | 233.788742 | 164.941787 | 0 | 0 | 54.980596 | 275.023501 | -2.301792 | 0.038828 |
| circRNA3555 | hsa_circ_0008750 | STAG1 | 2.543242 | 1.967973 | 2.589006 | 0 | 0 | 0 | 0 | 2.36674 | -1.751352 | 0.007053 |
| circRNA3670 | novel_circ | LINC00299 | 103.483626 | 82.339681 | 77.066535 | 38.985422 | 26.728831 | 0 | 21.904751 | 87.629947 | -1.952147 | 0.01231 |
| circRNA3865 | hsa_circ_0055499 | PTCD3 | 6.542255 | 9.887116 | 11.199422 | 0 | 0 | 0 | 0 | 9.209598 | -3.351854 | 0.02192 |
| circRNA3985 | hsa_circ_0057105 | PDK1 | 27.290795 | 30.983041 | 18.150955 | 11.343063 | 0 | 0 | 3.781021 | 25.47493 | -2.469236 | 0.015613 |
| circRNA3997 | hsa_circ_0004305 | TTN-AS1 | 14.442032 | 10.805955 | 9.726101 | 0 | 0 | 0 | 0 | 11.658029 | -3.661981 | 0.014644 |
| circRNA4102 | hsa_circ_0058876 | ILKAP | 19.022791 | 20.93608 | 20.318029 | 0 | 0 | 0 | 0 | 20.0923 | -4.398645 | 0.000786 |
| circRNA4236 | hsa_circ_0004893 | PTP4A2 | 25.93995 | 21.403372 | 30.088364 | 0 | 0 | 0 | 0 | 25.810562 | -4.74473 | 0.00931 |
| circRNA4320 | novel_circ | ALG6 | 10.923796 | 10.11733 | 7.527732 | 0 | 0 | 0 | 0 | 9.522953 | -3.395468 | 0.011375 |
| circRNA4487 | hsa_circ_0014615 | DAP3 | 14.109965 | 15.89605 | 12.798216 | 0 | 0 | 0 | 0 | 14.268077 | -3.932446 | 0.003936 |
| circRNA4711 | hsa_circ_0003410 | UBAP2 | 3.926232 | 4.429286 | 4.780181 | 1.782137 | 1.931764 | 0 | 1.237967 | 4.378567 | -1.265033 | 0.02445 |
| circRNA4713 | novel_circ | TLN1 | 62.235302 | 36.349622 | 54.301551 | 0 | 0 | 0 | 0 | 50.962158 | -5.699389 | 0.021837 |
| circRNA4947 | novel_circ | MYBL1 | 13.232312 | 12.70748 | 16.132952 | 0 | 8.651843 | 0 | 2.883948 | 14.024248 | -1.951697 | 0.047459 |
| circRNA582 | novel_circ | PPP6R3 | 14.214976 | 14.513933 | 13.463728 | 7.035788 | 6.453103 | 0 | 4.496297 | 14.064212 | -1.454593 | 0.048868 |
| circRNA741 | hsa_circ_0000211 | SFMBT2 | 9.93897 | 8.664516 | 9.430717 | 4.045043 | 3.578706 | 3.741158 | 3.788302 | 9.344734 | -1.11131 | 0.001794 |
| circRNA785 | hsa_circ_0005125 | YME1L1 | 5.085494 | 5.548191 | 6.313409 | 0 | 0 | 0 | 0 | 5.649031 | -2.733144 | 0.003993 |
| circRNA828 | hsa_circ_0018401 | SGMS1 | 163.92929 | 168.44795 | 139.82228 | 74.894995 | 41.105181 | 75.264814 | 63.754997 | 157.39984 | -1.290507 | 0.003481 |
| circRNA874 | hsa_circ_0018998 | CCSER2 | 125.938546 | 141.036987 | 128.119949 | 43.659083 | 35.084142 | 0 | 26.247741 | 131.698494 | -2.283943 | 0.009222 |
| circRNA960 | hsa_circ_0005418 | CTBP2 | 145.230289 | 131.357784 | 165.495017 | 58.846662 | 18.903277 | 0 | 25.916646 | 147.36103 | -2.462542 | 0.007536 |
