# Supplementary material for: Comprehensive analysis of circRNA expression profiles and circRNA-associated competing endogenous RNA networks in IgA nephropathy
Source: PeerJ. 2020 Dec 3;8:e10395. doi: 10.7717/peerj.10395 (PMC7719294; doi:10.7717/peerj.10395)
Supplement: Supplemental Information 4 [file peerj-08-10395-s004.docx]

**Table S3. Dysregulated miRNA transcripts between controls and IgAN patients.**

| **miRNA** | **Control** | **IgAN** | **log_2_(FC)** | **P-value** | **q value** |
| --- | --- | --- | --- | --- | --- |
| hcmv-miR-UL70-3p | 3.215384021 | 4.51673461 | 1.301350588 | 0.015044985 | 0.042577307 |
| hcmv-miR-US4 | 2.125795188 | 3.598526284 | 1.472731096 | 0.000962352 | 0.009113132 |
| hsa-miR-1224-5p | 4.239755808 | 5.31887612 | 1.079120312 | 0.000489018 | 0.006988488 |
| hsa-miR-193a-5p | 2.620428279 | 4.165191715 | 1.544763435 | 6.76E-06 | 0.001911693 |
| hsa-miR-22-3p | 3.44147954 | 4.645225586 | 1.203746045 | 0.001010499 | 0.009224875 |
| hsa-miR-23a-3p | 3.415181862 | 4.651916105 | 1.236734242 | 0.000323314 | 0.005724738 |
| hsa-miR-518c-3p | 1.791298292 | 3.32604379 | 1.534745498 | 0.000197226 | 0.004293456 |
| hsa-miR-584-5p | 2.044861763 | 3.293932311 | 1.249070548 | 0.00455781 | 0.018584921 |
| hsa-miR-601 | 2.080354972 | 3.543536638 | 1.463181666 | 0.000609015 | 0.006988488 |
| hsa-miR-623 | 0.931868555 | 2.752026321 | 1.820157767 | 0.002917962 | 0.013319083 |
| hsa-miR-629-5p | 2.735298365 | 3.755249991 | 1.019951626 | 0.004726335 | 0.018584921 |
| hsa-miR-654-5p | 0.871672549 | 2.569967081 | 1.698294532 | 0.004728319 | 0.018584921 |
| hsa-miR-671-5p | 3.782419832 | 4.960456411 | 1.178036579 | 0.000106924 | 0.004191302 |
| hsa-miR-760 | 1.823796894 | 3.643411699 | 1.819614805 | 5.02E-05 | 0.002839819 |
| hsa-miR-765 | 3.820714093 | 4.894083054 | 1.073368962 | 0.007294004 | 0.025483991 |
| hsa-miR-923 | 12.5759092 | 13.65455649 | 1.078647282 | 0.01900571 | 0.049801998 |
| hsa-miR-93-3p | 0.615909918 | 1.743883596 | 1.127973678 | 0.008054425 | 0.026200025 |
| hsv1-miR-H1 | 1.217132373 | 3.959726719 | 2.742594346 | 0.000696268 | 0.007578609 |
| hsa-miR-212-5p | 5.88256842 | 4.50094286 | -1.38162556 | 2.23E-05 | 0.00210704 |
| hsa-miR-30a-5p | 5.448028715 | 3.935097167 | -1.512931548 | 4.90E-05 | 0.002839819 |
| hsa-miR-650 | 3.166816102 | 1.180740201 | -1.986075901 | 0.000343889 | 0.005724738 |
| kshv-miR-K12-10a-5p | 2.892512261 | 1.874811114 | -1.017701148 | 0.001759381 | 0.010578511 |
